# The International Medical Congress

**Published:** 1887-07

**Authors:** 


### EDITORIAL.

### THE INTERNATIONAL MEDICAL CONGRESS.

The latest reports that are reliable go to show that the scientific work of the Congress will be of a high order. Nothing can prevent a grand success, scientifically speaking. We are informed that a sufficient number of papers have already been promised by members from abroad to occupy the whole time of each section. This is surely a good endorsement of the Congress. If the above statement is true, it follows of necessity that a large number of foreign physicians and their families will visit this country in September; and, while ostensibly they come on purely scientific business, yet much will be expected in a social way.

The time has come when it is necessary that large amounts of money be placed at the disposal of the management. As already said, from a scientific standpoint the congress will be a decided success; but we admit with much reluctance the fact that when the pocket-book is tapped the outflow is extremely small. Some of the state and local societies have done their share in the way of contributions. Illinois has contributed $750, and many other states equally well. But in some portions of the country the available amounts have been very small. In this connection we cannot but regret the action taken at the last meeting of the American Medical Association. For an association which invited the Congress to visit this country, and which is to act as host to the visiting members, to contribute the paltry sum of one thousand dollars seems ridiculous. No plea of lack of funds in the treasury is sufficient to excuse the association. It ought to have been the business of the officers to see that at least a respectable sum was kept in the treasury for the expenses of the Congress if the Association should direct its expenditure in that way.

With an income of over twenty thousand dollars, and with a journal which ought to pay a handsome revenue into the treasury, nothing short of a contribution of five thousand dollars would seem in proportion to the amounts contributed by state and local societies. Not having sufficient funds in the treasury to make a respectable contribution, it would have been a proper thing for the association to have levied a special assessment of, say, one dollar on each member. This would have furnished at least four thousand dollars.

The amounts needed must be made up by private contributions. The sooner these are forwarded the more good will they do. Let every one give something, and let it be done speedily and with good cheer. The profession in America cannot afford to neglect the proper entertainment of its guests, and must do individually what its representative body has failed to do.

